# Survival impact of additional chemotherapy after adjuvant concurrent chemoradiation in patients with early cervical cancer who underwent radical hysterectomy

**DOI:** 10.1186/s12885-021-08940-z

**Published:** 2021-11-22

**Authors:** Se Ik Kim, Jeong Yun Kim, Chan Woo Wee, Maria Lee, Hee Seung Kim, Hyun Hoon Chung, Taek Sang Lee, Hye Won Jeon, Noh Hyun Park, Yong Sang Song, Tae Hun Kim

**Affiliations:** 1 Department of Obstetrics and Gynecology, Seoul National University College of Medicine, 101, Daehak-ro, Jongno-gu, Seoul, 03080 Republic of Korea; 2 Department of Radiation Oncology, Seoul Metropolitan Government Seoul National University Boramae Medical Center, 20, Boramae-ro 5-gil, Dongjak-gu, Seoul, 07061 Republic of Korea; 3 Department of Obstetrics and Gynecology, Seoul Metropolitan Government Seoul National University Boramae Medical Center, 20, Boramae-ro 5-gil, Dongjak-gu, Seoul, 07061 Republic of Korea

**Keywords:** Uterine cervical neoplasms, Hysterectomy, Chemoradiotherapy, Chemotherapy, adjuvant, Prognosis

## Abstract

**Background:**

To determine whether additional chemotherapy after concurrent chemoradiation (CCRT) improves survival outcomes in patients with early cervical cancer who undergo radical hysterectomy (RH).

**Methods:**

We included high- or intermediate-risk patients from two institutions, with 2009 FIGO stage IB–IIA, who underwent primary RH and pelvic lymphadenectomy between January 2007 and June 2020, and had completed adjuvant CCRT. Survival outcomes were compared between patients who received additional chemotherapy (study group) and those who did not (control group).

**Results:**

A total of 198 patients were included in this analysis. The study (*n* = 61) and control groups (*n* = 137) had similar patient age, histologic cancer type, 2009 FIGO stage, and tumor size. However, minimally invasive surgery was performed less frequently in the study group than in the control group (19.7% vs. 46.0%, *P* < 0.001). The presence of pathologic risk factors was similar, except for lymph node metastasis, which was more frequent in the study group (72.1% vs. 46.0%; *P* = 0.001). In survival analyses, no differences in the disease-free survival (DFS; *P* = 0.539) and overall survival (OS; *P* = 0.121) were observed between the groups. Multivariate analyses adjusting for surgical approach and other factors revealed that additional chemotherapy was not associated with DFS (adjusted HR, 1.149; 95% CI, 0.552–2.391; *P* = 0.710) and OS (adjusted HR, 1.877; 95% CI, 0.621–5.673; *P* = 0.264). The recurrence patterns did not differ with additional chemotherapy. Consistent results were observed in a subset of high-risk patients (*n* = 139).

**Conclusions:**

Additional chemotherapy after CCRT might not improve survival outcomes in patients with early cervical cancer who undergo RH.

**Supplementary Information:**

The online version contains supplementary material available at 10.1186/s12885-021-08940-z.

## Background

Cervical cancer is a global burden, as it ranks the fourth in new cancer cases and is the leading cause of cancer-related death in women [1]. The incidence and mortality rates of cervical cancer show geographic discrepancies. Although there was a definitive decrease in the incidence of cervical cancer owing to the national screening program and human papillomavirus vaccination [2], the age-standardized incidence of cervical cancer in Korea is much higher than that in the United States (13.5 vs. 2.2 per 100,000 in 2017) [3, 4]. Primary radical hysterectomy (RH) and pelvic lymphadenectomy are the current standard of care for early invasive cervical cancer [5, 6].

After surgery, adjuvant pelvic radiation therapy (RT) with concurrent platinum-containing chemotherapy is recommended in patients who have at least one of the following three high-risk factors: positive lymph nodes (LNs), parametrial invasion, and positive resection margins [5, 7]. Adjuvant RT with or without concurrent chemotherapy is also recommended in node-negative, margin-negative, and parametria-negative patients, if they have intermediate risk factors that meet the Sedlis criteria [5, 7, 8]. Moreover, some physicians often prescribe sequential additional chemotherapy to reduce disease recurrence and especially the development of distant metastases.

The exact role of additional chemotherapy on survival outcomes is still not fully understood. Moreover, additional chemotherapy after concurrent chemoradiation therapy (CCRT) might cause additional complications, such as peripheral neuropathy and hematologic, gastrointestinal, or renal toxicities, and increases costs. Currently, RTOG-0724 (NCT00980954), a phase III randomized controlled trial (RCT) of patients with high-risk early-stage cervical cancer undergoing RH with CCRT or CCRT plus additional chemotherapy, is in progress. The primary endpoint of RTOG-0724 is disease-free survival (DFS). However, it is expected to take several years until the study results are announced.

Thus, this study aimed to determine whether additional chemotherapy after CCRT improves survival outcomes in patients with high- or intermediate-risk early-stage cervical cancer who underwent RH. As the Laparoscopic Approach to Cervical Cancer (LACC) trial, a phase III RCT on surgical approach in early cervical cancer, identified minimally invasive RH as a risk factor for disease recurrence and mortality [9], we adjusted the surgical approach as an important confounder.

## Methods

### Study population

From the cervical cancer cohort of the two institutions, patients with 2009 International Federation of Gynecology and Obstetrics (FIGO) stage IB1–IIA2 were identified [10]. We included patients who met the following criteria: (1) underwent primary Querleu-Morrow Type C RH [11] and pelvic lymphadenectomy by either open surgery or minimally invasive surgery (MIS) between January 2007 and June 2020; (2) had squamous cell carcinoma, usual type adenocarcinoma, or adenosquamous carcinoma; (3) pathologically proven high-risk or intermediate-risk group, as defined by the Sedlis criteria [8] (positive lymphovascular space invasion (LVSI) and deep third stromal invasion; positive LVSI, middle third stromal invasion, and cervical tumor size ≥2 cm; positive LVSI, superficial third stromal invasion, and cervical tumor size ≥5 cm; and negative LVSI, middle or deep third stromal invasion, and cervical tumor size ≥4 cm); and (4) completed adjuvant CCRT in accordance with the current practice guidelines [12]. Patients were excluded if: (1) the surgery was performed by inexperienced surgeons (i.e., fellows); (2) they received neoadjuvant chemotherapy or RT before surgery; (3) they were enrolled in first-line clinical trials, such as RTOG-0724, GOG-0263 (NCT01101451), and SHAPE (NCT01658930); and (4) they were lost to follow-up during primary treatment, or had insufficient clinicopathological data. Based on these criteria, 198 patients were selected as the study population.

### Data collection

Patient clinicopathologic characteristics, including age, surgical approach, conization, histologic type, 2009 FIGO stage, para-aortic lymphadenectomy, extent of invasion, risk factors for recurrence, and detailed information on adjuvant treatment, were gathered through a review of medical records. For the clinical tumor size, we retrieved the tumor size, either examined under colposcopy or measured using preoperative magnetic resonance imaging (MRI). For the pathologic tumor size, we retrieved the tumor size measured from the uterine specimens. We also collected the presence or absence of gastrointestinal toxicities and severity of hematologic toxicities that occurred during adjuvant treatment according to the Common Terminology Criteria for Adverse Events version 5.0 [13].

RH was performed by eight gynecologic oncology faculties from the two hospitals. Before the reports from the LACC trial [9], no internal policies existed on selecting patients to undergo MIS RH or open RH in early-stage cervical cancer; the surgical approach was chosen according to the surgeon’s preference. After the LACC trial, all patients were informed of the risks associated with MIS RH. Based on the results of our retrospective study, patients with a tumor size > 20 mm on preoperative MRI underwent open RH [14].

We administered pelvic external beam RT (EBRT) 45.0–50.4 Gy in 25–28 fractions to the patients. EBRT planning and delivery methods changed from 3D conformal RT to intensity-modulated RT (IMRT) in July 2015 and November 2015 at Seoul National University Boramae Medical Center and Seoul National University Hospital, respectively. Extended-field RT was delivered in patients with pathologically confirmed common iliac and/or para-aortic LN metastasis. The dose prescribed to the para-aortic region was 45.0 Gy in 25 fractions, and boost treatment was conducted in patients suspicious of residual LN metastasis. In patients with surgical resection margins < 5 mm or initially bulky tumors, intracavitary radiotherapy (ICR) 15 Gy in 3 fractions was administered following pelvic EBRT at the discretion of the radiation oncologists. During RT, which usually took 5–6 weeks, patients received one of the following chemotherapy regimens concomitantly: (1) cisplatin 40 mg/m^2^ weekly; (2) cisplatin 75 mg/m^2^ every three weeks for two cycles; (3) paclitaxel 135 mg/m^2^ plus carboplatin dose of AUC 5.0 every three weeks for two cycles; or (4) 5-fluorouracil (5FU) 1000 mg/m^2^ plus cisplatin 60 mg/m^2^ every three weeks for two cycles.

After completion of CCRT, some physicians recommended the following additional chemotherapy regimens to patients: (1) cisplatin 40 mg/m^2^ weekly; (2) cisplatin 75 mg/m^2^ every three weeks; (3) paclitaxel 175 mg/m^2^ plus carboplatin dose of AUC 5.0 every three weeks; or (4) 5FU 1000 mg/m^2^ plus cisplatin 60 mg/m^2^ every three weeks. Regardless of additional chemotherapy regimens, the physicians decided to prescribe another three or six cycles based on computed tomography (CT) scans obtained within one month after the initial three cycles of the additional chemotherapy.

Patients underwent surveillance with CT scans every three–four months for the first two years, every six months for the next two years, and annually thereafter. DFS and overall survival (OS) were defined as the time interval from the date of RH to the date of disease progression based on the Response Evaluation Criteria in Solid Tumors version 1.1 [15] and cancer-related death or the end of the study, respectively. The recurrence site in each patient was recorded according to anatomic sites from the CT scans obtained at the time of disease progression. In terms of recurrence patterns, we categorized patients into local failure (i.e., recurrence in the vaginal stump, pelvic wall, pelvic peritoneum and organs, or pelvic LNs), distant failure (i.e., recurrence in the abdominal peritoneum and organs, para-aortic LNs, supraclavicular LNs, lung, mediastinum, or bone), or both.

### Statistical analysis

The study population was divided into two groups according to the administration of additional chemotherapy. We compared the patient characteristics and survival outcomes between the groups. We used the Student’s t-test or Mann-Whitney U-test for continuous variables and Pearson’s chi-square test or Fisher’s exact test for categorical variables. For survival analyses, the Kaplan–Meier method with the log-rank test was used. In the multivariate analysis, Cox proportional hazards regression models were used to calculate adjusted hazard ratios (aHRs) and 95% confidence intervals (CIs). All statistical analyses were performed using the SPSS statistical software (version 25.0; IBM Corp. Armonk, NY, USA). Statistical significance was set at *P* < 0.05.

## Results

### Analysis in all patients

Of the 198 patients, 61 (30.8%) received additional chemotherapy (study group), while 137 (69.2%) did not (control group). Table 1 presents the patients’ clinicopathologic characteristics. Patient age, proportion of preoperative conization, histologic type, tumor size, and 2009 FIGO stage were similar between the two groups. However, the study group received MIS RH less frequently (19.7% vs. 46.0%; *P* < 0.001) and had a larger number of removed pelvic LNs (median, 29 vs. 23; *P* = 0.001) than the control group. Para-aortic lymphadenectomy was performed more frequently in the study group with borderline statistical significance (41.0% vs. 27.0%; *P* = 0.050), but the number of removed LNs were similar between the study and control groups (*P* = 0.235). Among the patients who did not receive para-aortic lymphadenectomy (*n* = 136), none was suspicious of para-aortic LN metastasis on preoperative imaging studies. Pathologic LN metastasis was more commonly identified in the study group (72.1% vs. 46.0%; *P* = 0.001). Accordingly, high-risk patients were more common in the study group than in the control group (85.2% vs. 63.5%; *P* = 0.002).

**Table 1:** Clinicopathologic characteristics of study population

Characteristics	Control group (***n*** = 137, %)	Study group (***n*** = 61, %)	***P***
Age, years			
Median (IQR)	53.1 (43.6–61.6)	49.1 (40.6–59.1)	0.095
BMI, kg/m^2^			
Mean ± SD	23.5 ± 4.0	24.2 ± 3.5	0.204
Surgical approach			0.002
Open	74 (54.0)	49 (80.3)	
Laparoscopy	53 (38.7)	9 (14.8)	
Robot-assisted surgery	10 (7.3)	3 (4.9)	
Conization	22 (16.1)	12 (19.7)	0.534
Histologic type			0.858
Squamous cell carcinoma	112 (81.8)	48 (78.7)	
Adenocarcinoma	20 (14.6)	10 (16.4)	
Adenosquamous carcinoma	5 (3.6)	3 (4.9)	
2009 FIGO stage			0.254
IB1	70 (51.1)	29 (47.5)	
IB2	32 (23.4)	12 (19.7)	
IIA1	13 (9.5)	12 (19.7)	
IIA2	22 (16.1)	8 (13.1)	
Pelvic lymphadenectomy			N/A
No	0	0	
Yes	137 (100.0)	61 (100.0)	
Removed LNs, median (IQR)	23 (16–31)	29 (21–35)	0.001
Positive LNs, median (IQR)	0 (0–2)	1 (0–4)	< 0.001
Para-aortic lymphadenectomy			0.050
No	100 (73.0)	36 (59.0)	
Yes	37 (27.0)	25 (41.0)	
Removed LNs, median (IQR)	3 (2–5.5)	4 (2.5–6)	0.235^a^
Positive LNs, median (IQR)	0 (0–0)	0 (0–1)	0.004^a^
Clinical cervical tumor size^*^, mm			
Mean ± SD	34.8 ± 13.9	31.9 ± 13.9	0.163
Pathologic cervical tumor size^†^, mm			
Median (IQR)	41.0 (30.5–56.0)	40.0 (30.0–51.5)	0.378
< 20	6 (4.4)	5 (8.2)	0.504
≥ 20 and < 40	56 (40.9)	23 (37.7)	
≥ 40 and < 50	26 (19.0)	15 (24.6)	
≥ 50	49 (35.8)	18 (29.5)	
Pathologic risk factors			
Parametrial invasion	46 (33.6)	25 (41.0)	0.316
LN metastasis	63 (46.0)	44 (72.1)	0.001
Pelvic LN only	62 (45.3)	37 (60.7)	0.008^b^
Both pelvic and para-aortic LNs	1 (0.7)	7 (11.5)	
Para-aortic LN only	0	0	
Resection margin involvement	15 (10.9)	9 (14.8)	0.449
LVSI	109 (79.6)	47 (77.0)	0.690
Deep stromal invasion	115 (83.9)	46 (75.4)	0.155
Indication for CCRT			0.002
High-risk factor	87 (63.5)	52 (85.2)	
Intermediate-risk factors	50 (36.5)	9 (14.8)	

Abbreviations: *CCRT* concurrent chemoradiation therapy; *FIGO* International Federation of Gynecology and Obstetrics; *IQR* interquartile range; *LN* lymph node; *LVSI* lymphovascular space invasion; *N/A* not applicable; *RT* radiation therapy; *SD* standard deviation

^*^Either examined under colposcopy or measured using preoperative magnetic resonance imaging

^†^Measured from the uterine specimen

^a^Among the patients who received para-aortic lymphadenectomy

^b^Among the patients with pathologic lymph node metastasis

In this study, all patients completed preplanned CCRT after surgery (Supplementary Table 1). While the use of ICR was similar between the study and control groups, extended field RT was performed more frequently in the study group (11.5% vs. 0.7%; *P* = 0.001). In terms of EBRT planning and delivery, IMRT was used less frequently in the study group than the control group (31.1% vs. 48.2%; *P* = 0.025). In the study group, paclitaxel plus carboplatin (49.2%) and weekly cisplatin (47.5%) were the two most common concomitant chemotherapy regimens, whereas weekly cisplatin was the most common (83.9%) in the control group. During CCRT, the study and control groups showed similar prevalence of gastrointestinal toxicities except diarrhea, which was more frequent in the study group (55.7% vs. 34.3%; *P* = 0.005).

Details of additional chemotherapy in the study group are shown in Supplementary Table 2. The most common additional chemotherapy regimen was paclitaxel plus carboplatin (59.0%), followed by 5FU plus cisplatin (32.8%), weekly cisplatin (6.6%), and triweekly cisplatin (1.6%). Of 61 patients, 16 (26.2%) refused scheduled additional chemotherapy cycles due to adverse events during additional chemotherapy. Overall, the most common hematologic toxicity during adjuvant treatment was anemia (89.4%), followed by neutropenia (65.7%), and both were more frequently observed in the study group than the control group (Supplementary Table 3). The study group had more grade 3/4 anemia, neutropenia, and thrombocytopenia. There was one septic shock case requiring admission to an intensive care unit in the study group. However, no mortality was observed during additional chemotherapy.

**Fig. 1:**
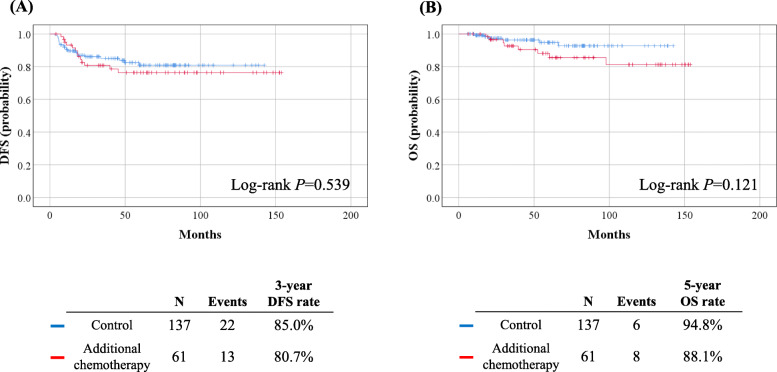
Comparisons of survival outcomes between study and control groups. (**A**) Disease-free survival; (**B**) Overall survival

During the median follow-up period of 53.8 months, 35 (17.7%) patients experienced disease recurrence, while 14 (7.1%) patients died of cervical cancer. In survival analyses, no differences in DFS (3-year DFS rate, 80.7% vs. 85.0%; *P* = 0.539) and OS (5-year OS rate, 88.1% vs. 94.8%; *P* = 0.121) were observed between the study and control groups (Fig. 1). In the multivariate analysis adjusted for histologic type, pathologic cervical tumor size, risk factors, and surgical approach, additional chemotherapy was not associated with DFS (aHR, 1.149; 95% CI, 0.552–2.391; *P* = 0.710) and OS (aHR, 1.877; 95% CI, 0.621–5.673; *P* = 0.264). Non-squamous cell carcinoma (*P* = 0.011), the presence of any high-risk factors (*P* = 0.043), and MIS RH (*P* = 0.030) were identified as poor prognostic factors for DFS, but not for OS (Table 2). In terms of recurrence sites and patterns, no differences were observed between the study and control groups (Supplementary Table 4).

**Table 2:** Univariate and multivariate analyses for survival outcomes in study population

Characteristics		*(A) Disease-free survival*						*(B) Overall survival*					
		Univariate analysis			Multivariate analysis			Univariate analysis			Multivariate analysis		
		HR	95% CI	*P*	aHR	95% CI	*P*	HR	95% CI	*P*	aHR	95% CI	*P*
Histology	Non-SCC vs. SCC	2.200	1.075–4.505	0.031	2.647	1.253–5.590	0.011	2.403	0.747–7.732	0.142	2.186	0.663–7.215	0.199
2009 FIGO stage	IIA vs. IB	0.860	0.403–1.835	0.696				1.876	0.650–5.416	0.245			
Para-aortic lymphadenectomy	Yes vs. No	1.090	0.542–2.190	0.809				1.394	0.482–4.034	0.540			
Pathologic cervical tumor size^*^	≥40 vs. < 40 mm	1.639	0.816–3.295	0.165	1.834	0.899–3.741	0.095	1.544	0.517–4.612	0.437	1.557	0.518–4.679	0.431
Indication for CCRT	High-risk vs. Low-risk	2.613	1.014–6.736	0.047	2.741	1.032–7.283	0.043	5.504	0.720–42.091	0.100	4.316	0.547–34.044	0.165
Surgical approach	MIS vs. Open surgery	1.667	0.859–3.239	0.131	2.178	1.079–4.397	0.030	0.798	0.247–2.580	0.706	1.132	0.333–3.850	0.842
Additional chemotherapy	Yes vs. No	1.240	0.624–2.461	0.539	1.149	0.552–2.391	0.710	2.270	0.782–6.584	0.131	1.877	0.621–5.673	0.264

Abbreviations: *CCRT* concurrent chemoradiation therapy; *FIGO* International Federation of Gynecology and Obstetrics

^*^Measured from the uterine specimen

### Subgroup analysis by risk groups

Subgroup analyses were conducted for high-risk patients (*n* = 139). In this subset, the study group had significantly younger patients (mean age, 50.0 vs. 53.9 years; *P* = 0.042), a smaller proportion of MIS RH (23.1% vs. 43.7%; *P* = 0.014), and a larger number of removed pelvic LNs (median, 28 vs. 23; *P* = 0.022), compared to the control group. Other baseline characteristics, including para-aortic lymphadenectomy, were similar between the two groups. Despite similar proportion of LN metastasis, among the patients with pathologic LN metastasis, pathologic para-aortic LN metastasis was more frequent in the study group (13.5% s. 1.1%; *P* = 0.008) (Table 3).

**Table 3:** Clinicopathologic characteristics of patients with high-risk factors

Characteristics	Control group (***n*** = 87, %)	Study group (***n*** = 52, %)	***P***
Age, years			
Mean ± SD	53.9 ± 11.3	50.0 ± 11.1	0.042
BMI, kg/m^2^			
Mean ± SD	23.1 ± 4.0	24.5 ± 3.6	0.054
Surgical approach			0.050
Open	49 (56.3)	40 (76.9)	
Laparoscopy	29 (33.3)	9 (17.3)	
Robot-assisted surgery	9 (10.3)	3 (5.8)	
Conization	11 (12.6)	10 (19.2)	0.294
Histologic type			0.297
Squamous cell carcinoma	74 (85.1)	39 (75.0)	
Adenocarcinoma	11 (12.6)	10 (19.2)	
Adenosquamous carcinoma	2 (2.3)	3 (5.8)	
2009 FIGO stage			0.699
IB1	42 (48.3)	24 (46.2)	
IB2	20 (23.0)	12 (23.1)	
IIA1	8 (9.2)	8 (15.4)	
IIA2	17 (19.5)	8 (15.4)	
Pelvic lymphadenectomy			N/A
No	0	0	
Yes	87 (100.0)	52 (100.0)	
Removed LNs, median (IQR)	23 (16–31.3)	28 (21.3–35)	0.022^a^
Positive LNs, median (IQR)	1 (0–3)	2 (1–5)	0.027^a^
Para-aortic lymphadenectomy			0.370
No	60 (69.0)	32 (61.5)	
Yes	27 (31.0)	20 (38.5)	
Removed LNs, median (IQR)	3 (1–5)	5 (3–6)	0.057
Positive LNs, median (IQR)	0 (0–0)	0 (0–2.5)	0.005
Clinical cervical tumor size^*^, mm			
Mean ± SD	35.2 ± 13.6	32.1 ± 14.7	0.191
Pathologic cervical tumor size^†^, mm			
Mean ± SD	45.6 ± 19.2	41.8 ± 19.6	0.262
< 20	5 (5.7)	5 (9.6)	0.681
≥ 20 and < 40	31 (35.6)	19 (36.5)	
≥ 40 and < 50	17 (19.5)	12 (23.1)	
≥ 50	34 (39.1)	16 (30.8)	
Pathologic risk factors			
Parametrial invasion	46 (52.9)	25 (48.1)	0.584
LN metastasis	63 (72.4)	44 (84.6)	0.098
Pelvic LN only	62 (71.3)	37 (71.2)	0.008^b^
Both pelvic and para-aortic LNs	1 (1.1)	7 (13.5)	
Para-aortic LN only	0	0	
Resection margin involvement	15 (17.2)	9 (17.3)	0.992
LVSI	71 (81.6)	40 (76.9)	0.505
Deep stromal invasion	73 (83.9)	39 (75.0)	0.199

Abbreviations: *CCRT* concurrent chemoradiation therapy; *FIGO* International Federation of Gynecology and Obstetrics; *IQR* interquartile range; *LN* lymph node; *LVSI* lymphovascular space invasion; *N/A* not applicable; *RT*, radiation therapy; *SD* standard deviation

^*^Either examined under colposcopy or measured using preoperative magnetic resonance imaging

^†^Measured from the uterine specimen

^a^Among the patients who received para-aortic lymphadenectomy

^b^Among the patients with pathologic lymph node metastasis

During CCRT, no differences in the use of IMRT and ICR were observed between the two groups, but extended field RT was performed more frequently in the study group (13.5% vs. 1.1%; *P* = 0.004) (Supplementary Table 5). Both paclitaxel plus carboplatin and weekly cisplatin were the most common concomitant chemotherapy regimens (48.1% for each) in the study group, whereas weekly cisplatin was the most common in the control group (79.3%). The two groups showed similar prevalence of gastrointestinal toxicities except diarrhea, which was more frequent in the study group (55.8% vs. 32.2%; *P* = 0.006).

Details of additional chemotherapy in the study group are shown in Supplementary Table 6. The most common additional chemotherapy regimen was paclitaxel plus carboplatin (59.6%), followed by 5FU plus cisplatin (34.6%), weekly cisplatin (3.8%), and triweekly cisplatin (1.9%). Of 52 patients, 13 (25.0%) refused scheduled additional chemotherapy cycles due to adverse events. Compared to the control group, the study group had more anemia (any grade), neutropenia (any grade), and thrombocytopenia (both any grade and grade 3/4) (Supplementary Table 7). No difference in grade 3/4 anemia was observed between the two groups. However, grade 3/4 neutropenia was marginally more frequent in the study group (15.4% vs. 4.6%; *P* = 0.056), but febrile neutropenia was similar between the two groups during adjuvant treatment.

**Fig. 2:**
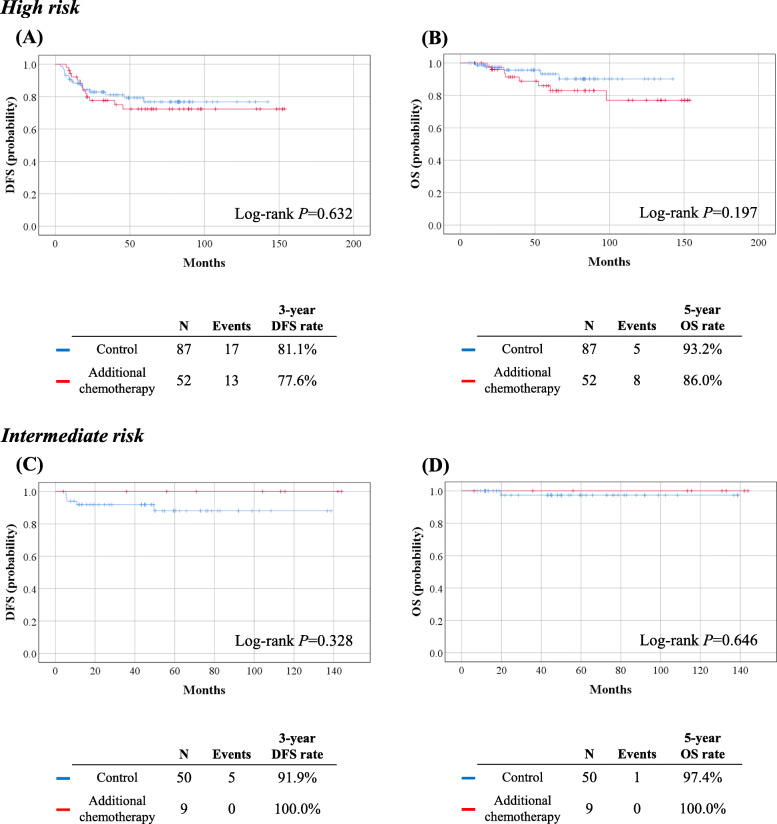
Survival outcomes stratified by risk factors. (Upper) Analysis in high-risk patients; (Lower) Analysis in intermediate-risk patiens. (**A**, **C**) Disease-free survival; (**B**, **D**) Overall survival

During the median follow-up period of 53.6 months, 30 (21.6%) patients experienced relapse, and 13 (9.4%) patients died of disease. In survival analyses, no differences in DFS (3-year DFS rate, 77.6% vs. 81.1%; *P* = 0.632) and OS (5-year OS rate, 86.0% vs. 93.2%; *P* = 0.197) were observed between the study and control groups (Fig. 2A, B). In the multivariate analysis adjusted for histologic type, pathologic cervical tumor size, risk factors, and surgical approach, additional chemotherapy was not associated with DFS (aHR, 1.138; 95% CI, 0.529–2.448; *P* = 0.742) or OS (aHR, 2.065; 95% CI, 0.622–6.857; *P* = 0.236). Parametrial invasion and LN metastasis were identified as poor prognostic factors for both DFS and OS. MIS RH was associated with significantly worse DFS (*P* = 0.002), but not OS (Table 4). The study and control groups showed similar recurrence sites and patterns (Supplementary Table 4).

**Table 4:** Univariate and multivariate analyses for survival outcomes in patients with high-risk factors

Characteristics		*(A) Disease-free survival*						*(B) Overall survival*					
		Univariate analysis			Multivariate analysis			Univariate analysis			Multivariate analysis		
		HR	95% CI	*P*	aHR	95% CI	*P*	HR	95% CI	*P*	aHR	95% CI	*P*
Histology	Non-SCC vs. SCC	2.040	0.932–4.464	0.074	2.230	0.961–5.175	0.062	1.658	0.453–6.060	0.445	1.246	0.321–4.827	0.751
2009 FIGO stage	IIA vs. IB	0.800	0.356–1.797	0.588				1.903	0.637–5.684	0.249			
Para-aortic lymphadenectomy	Yes vs. No	0.934	0.437–1.995	0.860				1.559	0.523–4.642	0.426			
Pathologic cervical tumor size^*^	≥40 vs. < 40 mm	1.368	0.651–2.874	0.409	1.514	0.692–3.309	0.299	1.716	0.528–5.578	0.369	1.289	0.368–4.515	0.691
Parametrial invasion	Yes vs. No	1.367	0.658–2.838	0.402	2.348	1.047–5.264	0.038	4.896	1.085–22.094	0.039	9.662	1.841–50.712	0.007
Lymph node metastasis	Yes vs. No	3.077	0.933–10.150	0.065	5.198	1.450–18.635	0.011	4.122	0.536–31.725	0.174	10.198	1.160–89.675	0.036
Surgical approach	MIS vs. Open surgery	2.072	1.011–4.247	0.047	3.406	1.558–7.448	0.002	1.030	0.312–3.400	0.961	3.029	0.775–11.833	0.111
Additional chemotherapy	Yes vs. No	1.193	0.579–2.456	0.632	1.138	0.529–2.448	0.742	2.062	0.671–6.335	0.206	2.065	0.622–6.857	0.236

Abbreviations: *CCRT* concurrent chemoradiation therapy; *FIGO* International Federation of Gynecology and Obstetrics. ^*^Measured from the uterine specimen

We also conducted subgroup analyses of intermediate-risk patients (*n* = 59). The median observation period for this subset was 53.9 months, during which five patients experienced relapse, and one patient died. Of the nine patients in the study group, none experienced disease recurrence or death. No significant differences in DFS (*P* = 0.328) and OS (*P* = 0.646) were observed between the study and control groups (Fig. 2C, D). Owing to the small sample size, we could not conduct further analyses.

## Discussion

In this study, we found that additional chemotherapy after CCRT did not improve survival outcomes in patients with 2009 FIGO stage IB1–IIA2 cervical cancer who underwent primary RH. Additional chemotherapy was associated with increased but clinically manageable hematologic toxicities. The recurrence sites and patterns did not differ, irrespective of whether additional chemotherapy was administered or not. These results were consistent with those in a subgroup of patients who had at least one of the three pathologic high-risk factors.

In fact, additional chemotherapy after CCRT is more common in locally advanced cervical cancer than in early-stage cervical cancer. For patients with 2009 FIGO stage IIB–IVA cervical cancer, phase III RCTs have compared survival outcomes between primary CCRT followed by additional chemotherapy versus primary CCRT alone with conflicting results [16–18]. While Dueñas-González et al. reported significantly improved DFS and OS from additional gemcitabine plus cisplatin chemotherapy [17], ACTLACC failed to prove survival benefit from additional paclitaxel plus carboplatin chemotherapy after CCRT [18]. Recently, results of a large phase III RCT, OUTBACK, have been reported [19]. In this large trial, which included a total of 926 patients with 2009 FIGO stage IB1 (LN positive) to IVA, no differences in DFS and OS, as well as patterns of disease recurrence were observed between the CCRT followed by four cycles of paclitaxel plus carboplatin chemotherapy arm and standard CCRT alone arm. For patients with early-stage cervical cancer who underwent primary surgery, studies investigating the role of additional chemotherapy after adjuvant CCRT are relatively insufficient despite its wide use in clinical practice.

Previously, Sun et al. conducted a phase III RCT in patients with intermediate-risk cervical cancer who underwent RH. To investigate the optimal adjuvant treatment in this group, patients were randomly assigned to three groups: RT only, CCRT, and CCRT followed by consolidation chemotherapy. However, this study was terminated early due to severe hematotoxicity in the CCRT plus chemotherapy group [20]. Zhao et al. conducted a phase III RCT in high-risk patients with early-stage cervical cancer who underwent RH. According to the interim analysis results of this study, CCRT plus additional chemotherapy did not improve DFS and OS compared to CCRT alone [21]. These results were consistent with those of our study.

Recently, a phase III RCT, named STARS, compared survival outcomes of different adjuvant treatment types—sequential chemoradiation, CCRT, and RT only—in patients with stage IB1–IIA2 cervical cancer who underwent RH and pelvic lymphadenectomy, and had adverse pathologic factors [22]. In this trial, sequential chemoradiation, consisting of each two cycles of paclitaxel plus cisplatin prior to and after RT, significantly improved DFS compared with CCRT (HR, 0.65; 95% CI, 0.44–0.96; *P* = 0.03) or RT alone (HR, 0.52; 95% CI, 0.35–0.76; *P* = 0.001), with tolerable toxic effects. However, sequential chemoradiation showed similar OS compared to CCRT (*P* = 0.25). Herein, we regarded the sequential chemoradiation of the STARS trial as the study group (CCRT followed by additional chemotherapy) in our study. Our results seem to be in line with the results extrapolated from the STARS trial.

We recognize that the appropriate adjuvant treatment of the patients who have intermediate-risk factors is still controversial. While Kim et al. conducted a retrospective study and reported that CCRT is not superior to RT alone after radical surgery in this group [23], a phase III RCT on this issue, GOG-263 (NCT01101451), is still ongoing. Nevertheless, to answer the key question of the current study, whether additional chemotherapy after adjuvant CCRT is beneficial or not, we included not only the high-risk group, but also the intermediate-risk group who underwent adjuvant CCRT, rather than RT alone. By confining the study population as those who received adjuvant CCRT after RH consistently, we could conduct fair comparisons in our study.

Before conducting this retrospective cohort study, we expected a survival benefit from additional chemotherapy. In contrast, additional chemotherapy failed to improve DFS or OS. One possible underlying mechanism is the presence of cancer stem cells (CSCs) and their acquisition of chemoresistance from the extended use of chemotherapy [24]. Gupta S. pointed out this issue in his editorial to the ACTLACC trial [25]: tumor clones that are resistant to or survive platinum-based CCRT are also resistant to further adjuvant platinum-based chemotherapy. In our study population, all visible tumors were removed by RH before the initiation of CCRT. However, CSCs may have remained within the tumor bed despite radical surgery, evolving towards resistant cells through postoperative adjuvant CCRT and additional chemotherapy [26, 27]. Such changes or evolution of CSCs might offset the cumulative cytotoxic effects from additional chemotherapy, resulting in no differences in survival outcomes.

In this study, we also investigated treatment-related adverse events of patients. In terms of gastrointestinal toxicities, the study group had more diarrhea than the control group, which might originate from the lower use of IMRT in the study group. Other gastrointestinal toxicities were similar between the two groups. Meanwhile, the study group experienced hematologic toxicities more frequently, compared to the control group. Moreover, a quarter of patients (26.2%) did not complete scheduled additional chemotherapy cycles due to adverse events. These findings were consistent with the OUTBACK [19]. Patients who receive additional chemotherapy might experience aggravation of toxicities from CCRT and experience toxicities from chemotherapy itself. In particular, the use of all three treatment modalities, surgery (RH plus pelvic lymphadenectomy), RT, and chemotherapy, definitely increases the incidence of adverse events. Therefore, development of a more individualized treatment strategy is necessary for high- or intermediate-risk early-stage cervical cancer. For example, if LN metastasis is detected intraoperatively, surgeons may abort scheduled RH based on the ABRAX study results [28]. If the ongoing RTOG-0724 trial proves no survival benefit from additional chemotherapy in high-risk patients with early-stage cervical cancer who underwent RH, additional chemotherapy may be omitted in this population. At the same time, an individualized approach, rather than an all-or-none approach, is necessary. By integrating an individual’s specific cliniocpathologic factors [29] and novel biomarkers predicting radiosensitivity or predisposition to RT-related toxicities [30], we might be able to develop tailored adjuvant treatment strategies. For example, the extent of radical surgery, RT field and methods, and additional chemotherapy regimens and cycles might be modulated. In accordance with the era of precision cancer medicine, further prospective studies are warranted.

Unlike previous studies, we included the surgical approach as a covariate in the multivariate analysis. The LACC trial revealed that MIS RH, rather than open RH, was associated with higher disease recurrence and mortality rates in early-stage cervical cancer [9]. Subsequent retrospective studies and a meta-analysis consistently reported inferior DFS from MIS RH [14, 31–33]. Similarly, in the current study, we identified MIS RH as an independent poor prognostic factor for DFS. However, MIS RH did not affect the OS. This finding is in line with the results of previous studies conducted by our research team [14]. The possible reasons for this might be the small sample size, short follow-up period, and few death events in the study population. In addition, we estimated that the patients who experienced relapse were successfully treated with subsequent therapies.

Our study has several limitations. First, selection bias might have been present owing to the retrospective study design. For example, the study and control groups showed differences in some baseline clinicopathologic characteristics, such as LN metastasis. Second, although this study was a bi-institutional study, the sample size was small, especially for intermediate-risk patients. If the sample size was sufficient, we could also conduct a subgroup analysis according to the surgical approach. Third, various chemotherapy regimens were administered during and after CCRT. In future, we may elucidate which chemotherapy regimen shows the highest efficacy. Lastly, not all toxicity profiles were investigated in this study. Quality of life issues were not compared between the study and control groups, either. Such information may enable further cost-effectiveness studies, which are very useful in clinical practice. Nevertheless, with clear inclusion and exclusion criteria and sequential multivariate analyses adjusting for confounders, we successfully showed no benefit from additional chemotherapy in a specific group of patients. We expect that the current RTOG-0724 trial will validate our study results.

## Conclusions

In conclusion, additional chemotherapy after CCRT was not associated with improved survival outcomes in patients with high- and intermediate-risk early-stage cervical cancer who underwent primary RH. Our study results suggest that additional chemotherapy after adjuvant CCRT is unnecessary, despite its frequent administration in clinical practice. Further studies with larger sample sizes are required to validate our results.

## Supplementary Information

**Additional file 1.**

**Additional file 2.**

**Additional file 3.**

**Additional file 4.**

**Additional file 5.**

**Additional file 6.**

**Additional file 7.**

## Data Availability

The datasets used and/or analyzed during the current study available from the corresponding author on reasonable request.

## Abbreviations

5FU: 5-fluorouracil
aHR: adjusted hazard ratio
CCRT: concurrent chemoradiation therapy
CI: confidence interval
CT: computed tomography
DFS: disease-free survival
EBRT: external beam radiation therapy
FIGO: International Federation of Gynecology and Obstetrics
ICR: intracavitary radiotherapy.
IMRT: intensity-modulated radiation therapy.
LACC: Laparoscopic Approach to Cervical Cancer.
LN: lymph node.
LVSI: lymphovascular space invasion.
MIS: minimally invasive surgery.
MRI: magnetic resonance imaging.
OS: overall survival.
RCT: randomized controlled trial.
RH: radical hysterectomy.
RT: radiation therapy.
