# Supplementary material for: Survival impact of additional chemotherapy after adjuvant concurrent chemoradiation in patients with early cervical cancer who underwent radical hysterectomy
Source: BMC Cancer. 2021 Nov 22;21:1260. doi: 10.1186/s12885-021-08940-z (PMC8609857; doi:10.1186/s12885-021-08940-z)
Supplement: Supplementary file 1 — Additional file 1. [file 12885_2021_8940_MOESM1_ESM.docx]

| **Supplementary Table 1.** CCRT methods and gastrointestinal toxicities in study population | | | |
| --- | --- | --- | --- |
| **Characteristics** | **Control group**  **(n=137, %)** | **Study group**  **(n=61, %)** | ***P*** |
| EBRT planning and delivery |  |  | 0.025 |
| 3D conformal RT | 71 (51.8) | 42 (68.9) |  |
| IMRT | 66 (48.2) | 19 (31.1) |  |
| Use of ICR |  |  | 0.930 |
| No | 113 (82.5) | 50 (82.0) |  |
| Yes | 24 (17.5) | 11 (18.0) |  |
| Use of extended field RT^*^ |  |  | 0.001 |
| No | 136 (99.3) | 54 (88.5) |  |
| Yes | 1 (0.7) | 7 (11.5) |  |
| Chemotherapy regimen during RT |  |  | <0.001 |
| Cisplatin, weekly | 115 (83.9) | 29 (47.5) |  |
| Cisplatin, tri-weekly | 10 (7.3) | 1 (1.6) |  |
| Paclitaxel-carboplatin | 9 (6.6) | 30 (49.2) |  |
| 5FU-cisplatin | 3 (2.2) | 1 (1.6) |  |
| Gastrointestinal toxicity, any grade^*^ |  |  |  |
| Nausea | 72 (52.6) | 33 (54.1) | 0.841 |
| Vomiting | 27 (19.7) | 13 (21.3) | 0.795 |
| Anorexia | 38 (27.7) | 22 (36.1) | 0.239 |
| Constipation | 41 (29.9) | 19 (31.1) | 0.863 |
| Diarrhea | 47 (34.3) | 34 (55.7) | 0.005 |
| Abbreviations: CCRT, concurrent chemoradiation therapy; EBRT, external beam radiation therapy; ICR, intracavitary radiotherapy; IMRT, intensity-modulated radiation therapy; RT, radiation therapy; 5FU, 5-fluorouracil. ^*^Common Terminology Criteria for Adverse Events (CTCAE) version 5.0. | | | |
