# Supplementary material for: Survival impact of additional chemotherapy after adjuvant concurrent chemoradiation in patients with early cervical cancer who underwent radical hysterectomy
Source: BMC Cancer. 2021 Nov 22;21:1260. doi: 10.1186/s12885-021-08940-z (PMC8609857; doi:10.1186/s12885-021-08940-z)
Supplement: Supplementary file 2 — Additional file 2. [file 12885_2021_8940_MOESM2_ESM.docx]

| **Supplementary Table 2.** Additional chemotherapy administration in study population | |
| --- | --- |
| **Characteristics** | **Study group**  **(n=61, %)** |
| ***Weekly cisplatin during RT*** |  |
| Weekly cisplatin, 3 cycles | 4 (6.6) |
| Paclitaxel-carboplatin, 3 cycles | 2 (3.3) |
| Paclitaxel-carboplatin, 4 cycles^*^ | 1 (1.6) |
| Paclitaxel-carboplatin, 6 cycles | 3 (4.9) |
| 5FU-cisplatin, 3 cycles | 10 (16.4) |
| 5FU-cisplatin, 4 cycles^*^ | 1 (1.6) |
| 5FU-cisplatin, 6 cycles | 7 (11.5) |
| 5FU-cisplatin, 9 cycles | 1 (1.6) |
| ***Tri-weekly cisplatin during RT*** |  |
| Triweekly cisplatin, 3 cycles | 1 (1.6) |
| ***Paclitaxel-carboplatin during RT*** |  |
| Paclitaxel-carboplatin, 2 cycles^*^ | 3 (4.9) |
| Paclitaxel-carboplatin, 3 cycles | 17 (27.9) |
| Paclitaxel-carboplatin, 4 cycles^*^ | 10 (16.4) |
| ***5FU-cisplatin during RT*** |  |
| 5FU-cisplatin, 4 cycles^*^ | 1 (1.6) |
| Abbreviations: RT, radiation therapy; 5FU, 5-fluorouracil. ^*^All 16 patients refused scheduled chemotherapy cycles due to adverse events during additional chemotherapy. | |
