# Supplementary material for: Survival impact of additional chemotherapy after adjuvant concurrent chemoradiation in patients with early cervical cancer who underwent radical hysterectomy
Source: BMC Cancer. 2021 Nov 22;21:1260. doi: 10.1186/s12885-021-08940-z (PMC8609857; doi:10.1186/s12885-021-08940-z)
Supplement: Supplementary file 3 — Additional file 3. [file 12885_2021_8940_MOESM3_ESM.docx]

| **Supplementary Table 3.** Hematologic toxicities and other adverse events in study population | | | |
| --- | --- | --- | --- |
| **Characteristics** | **Control group**  **(n=137, %)** | **Study group**  **(n=61, %)** | ***P*** |
| Neutropenia^*^ |  |  |  |
| Any grade | 71 (51.8) | 59 (96.7) | <0.001 |
| Grade ≥3 | 19 (13.9) | 42 (68.9) | <0.001 |
| Anemia^*^ |  |  |  |
| Any grade | 116 (84.7) | 61 (100.0) | 0.001 |
| Grade ≥3 | 6 (4.4) | 10 (16.4) | 0.009 |
| Thrombocytopenia^*^ |  |  |  |
| Any grade | 50 (36.5) | 31 (50.8) | 0.058 |
| Grade ≥3 | 2 (1.5) | 5 (8.2) | 0.030 |
| Other adverse events |  |  |  |
| Febrile neutropenia | 2 (1.5) | 2 (3.3) | 0.588 |
| Septic shock | 0 | 1 (1.6) | 0.308 |
| ICU admission | 0 | 1 (1.6) | 0.308 |
| Death | 0 | 0 | N/A |
| Abbreviations: ICU, intensive care unit; N/A, not applicable. ^*^Common Terminology Criteria for Adverse Events (CTCAE) version 5.0. | | | |
