# Supplementary material for: Survival impact of additional chemotherapy after adjuvant concurrent chemoradiation in patients with early cervical cancer who underwent radical hysterectomy
Source: BMC Cancer. 2021 Nov 22;21:1260. doi: 10.1186/s12885-021-08940-z (PMC8609857; doi:10.1186/s12885-021-08940-z)
Supplement: Supplementary file 4 — Additional file 4. [file 12885_2021_8940_MOESM4_ESM.docx]

**Supplementary Table 4.** Recurrence sites and patterns of patients

|  | ***All patients*** | | | ***High-risk*** | | | ***Intermediate-risk*** | | |
| --- | --- | --- | --- | --- | --- | --- | --- | --- | --- |
|  | **Control group**  **(n=137, %)** | **Study group**  **(n=61, %)** | ***P*** | **Control group**  **(n=87, %)** | **Study group**  **(n=52, %)** | ***P*** | **Control group**  **(n=50, %)** | **Study group**  **(n=9, %)** | ***P*** |
| Total recurrence | 22 (16.1) | 13 (21.3) | 0.371 | 17 (19.5) | 13 (25.0) | 0.449 | 5 (10.0) | 0 | >0.999 |
| Recurrence sites |  |  |  |  |  |  |  |  |  |
| Vaginal stump | 6 (4.4) | 4 (6.6) | 0.501 | 4 (4.6) | 4 (7.7) | 0.472 | 2 (4.0) | 0 | >0.999 |
| Pelvic wall | 2 (1.5) | 1 (1.6) | >0.999 | 2 (2.3) | 1 (1.9) | >0.999 | 0 | 0 | N/A |
| Pelvic peritoneum and organ | 6 (4.4) | 3 (4.9) | >0.999 | 6 (6.9) | 3 (5.8) | >0.999 | 0 | 0 | N/A |
| Pelvic LN | 5 (3.6) | 0 | 0.326 | 4 (4.6) | 0 | 0.297 | 1 (2.0) | 0 | >0.999 |
| Abdominal peritoneum and organ | 6 (4.4) | 4 (6.6) | 0.501 | 5 (5.7) | 4 (7.7) | 0.728 | 1 (2.0) | 0 | >0.999 |
| Para-aortic LN | 8 (5.8) | 2 (3.3) | 0.727 | 8 (9.2) | 2 (3.8) | 0.321 | 0 | 0 | N/A |
| Supraclavicular LN | 1 (0.7) | 1 (1.6) | 0.522 | 1 (1.1) | 1 (1.9) | >0.999 | 0 | 0 | N/A |
| Lung | 6 (4.4) | 3 (4.9) | >0.999 | 3 (3.4) | 3 (5.8) | 0.671 | 3 (6.0) | 0 |  |
| Mediastinum | 1 (0.7) | 1 (1.6) | 0.522 | 1 (1.1) | 1 (1.9) | >0.999 | 0 | 0 | N/A |
| Bone | 1 (0.7) | 1 (1.6) | 0.522 | 1 (1.1) | 1 (1.9) | >0.999 | 0 | 0 | N/A |
| Recurrence patterns |  |  |  |  |  |  |  |  |  |
| Local failure | 13 (9.5) | 6 (9.8) | 0.939 | 11 (12.6) | 6 (11.5) | 0.847 | 2 (4.0) | 0 | >0.999 |
| Distant failure | 17 (12.4) | 9 (14.8) | 0.652 | 13 (14.9) | 9 (17.3) | 0.712 | 4 (8.0) | 0 | >0.999 |
| Both | 8 (5.8) | 2 (3.3) | 0.727 | 7 (8.0) | 2 (3.8) | 0.484 | 1 (2.0) | 0 | >0.999 |
| Abbreviation: LN, lymph node; N/A, not applicable. | | | | | | | | | |
