# Supplementary material for: Survival impact of additional chemotherapy after adjuvant concurrent chemoradiation in patients with early cervical cancer who underwent radical hysterectomy
Source: BMC Cancer. 2021 Nov 22;21:1260. doi: 10.1186/s12885-021-08940-z (PMC8609857; doi:10.1186/s12885-021-08940-z)
Supplement: Supplementary file 5 — Additional file 5. [file 12885_2021_8940_MOESM5_ESM.docx]

| **Supplementary Table 5.** CCRT methods and gastrointestinal toxicities in patients with high-risk factors | | | |
| --- | --- | --- | --- |
| **Characteristics** | **Control group**  **(n=87, %)** | **Study group**  **(n=52, %)** | ***P*** |
| EBRT planning and delivery |  |  | 0.158 |
| 3D conformal RT | 48 (55.2) | 35 (67.3) |  |
| IMRT | 39 (44.8) | 17 (32.7) |  |
| Use of ICR |  |  | 0.744 |
| No | 70 (80.5) | 43 (82.7) |  |
| Yes | 17 (19.5) | 9 (17.3) |  |
| Use of extended field RT^*^ |  |  | 0.004 |
| No | 86 (98.9) | 45 (86.5) |  |
| Yes | 1 (1.1) | 7 (13.5) |  |
| Chemotherapy regimen during RT |  |  | <0.001 |
| Cisplatin, weekly | 69 (79.3) | 25 (48.1) |  |
| Cisplatin, tri-weekly | 9 (10.3) | 1 (1.9) |  |
| Paclitaxel-carboplatin | 6 (6.9) | 25 (48.1) |  |
| 5FU-cisplatin | 3 (3.4) | 1 (1.9) |  |
| Gastrointestinal toxicity, any grade^*^ |  |  |  |
| Nausea | 46 (52.9) | 29 (55.8) | 0.740 |
| Vomiting | 19 (21.8) | 11 (21.2) | 0.924 |
| Anorexia | 25 (28.7) | 18 (34.6) | 0.468 |
| Constipation | 25 (28.7) | 17 (32.7) | 0.623 |
| Diarrhea | 28 (32.2) | 29 (55.8) | 0.006 |
| Abbreviations: CCRT, concurrent chemoradiation therapy; EBRT, external beam radiation therapy; ICR, intracavitary radiotherapy; IMRT, intensity-modulated radiation therapy; RT, radiation therapy; 5FU, 5-fluorouracil.  ^*^Common Terminology Criteria for Adverse Events (CTCAE) version 5.0. | | | |
