# Supplementary material for: Survival impact of additional chemotherapy after adjuvant concurrent chemoradiation in patients with early cervical cancer who underwent radical hysterectomy
Source: BMC Cancer. 2021 Nov 22;21:1260. doi: 10.1186/s12885-021-08940-z (PMC8609857; doi:10.1186/s12885-021-08940-z)
Supplement: Supplementary file 6 — Additional file 6. [file 12885_2021_8940_MOESM6_ESM.docx]

| **Supplementary Table 6.** Additional chemotherapy administration in patients with high-risk factors | |
| --- | --- |
| **Characteristics** | **Study group**  **(n=52, %)** |
| ***Weekly cisplatin during RT*** |  |
| Weekly cisplatin, 3 cycles | 2 (3.8) |
| Paclitaxel-carboplatin, 3 cycles | 2 (3.8) |
| Paclitaxel-carboplatin, 4 cycles^*^ | 1 (1.9) |
| Paclitaxel-carboplatin, 6 cycles | 3 (5.8) |
| 5FU-cisplatin, 3 cycles | 8 (15.4) |
| 5FU-cisplatin, 4 cycles^*^ | 1 (1.9) |
| 5FU-cisplatin, 6 cycles | 7 (13.5) |
| 5FU-cisplatin, 9 cycles | 1 (1.9) |
| ***Tri-weekly cisplatin during RT*** |  |
| Triweekly cisplatin, 3 cycles | 1 (1.9) |
| ***Paclitaxel-carboplatin during RT*** |  |
| Paclitaxel-carboplatin, 2 cycles^*^ | 2 (3.8) |
| Paclitaxel-carboplatin, 3 cycles | 15 (28.8) |
| Paclitaxel-carboplatin, 4 cycles^*^ | 8 (15.4) |
| ***5FU-cisplatin during RT*** |  |
| 5FU-cisplatin, 4 cycles^*^ | 1 (1.9) |
| Abbreviations: RT, radiation therapy; 5FU, 5-fluorouracil. *All 13 patients refused scheduled chemotherapy cycles due to adverse events during additional chemotherapy. | |
