# Supplementary material for: Survival impact of additional chemotherapy after adjuvant concurrent chemoradiation in patients with early cervical cancer who underwent radical hysterectomy
Source: BMC Cancer. 2021 Nov 22;21:1260. doi: 10.1186/s12885-021-08940-z (PMC8609857; doi:10.1186/s12885-021-08940-z)
Supplement: Supplementary file 7 — Additional file 7. [file 12885_2021_8940_MOESM7_ESM.docx]

| **Supplementary Table 7.** Hematologic toxicities and other adverse events in patients with high-risk factors | | | |
| --- | --- | --- | --- |
| **Characteristics** | **Control group**  **(n=87, %)** | **Study group**  **(n=52, %)** | ***P*** |
| Neutropenia^*^ |  |  |  |
| Any grade | 41 (47.1) | 50 (96.2) | <0.001 |
| Grade ≥3 | 4 (4.6) | 8 (15.4) | 0.056 |
| Anemia^*^ |  |  |  |
| Any grade | 70 (80.5) | 52 (100.0) | 0.001 |
| Grade ≥3 | 2 (2.3) | 3 (5.8) | 0.363 |
| Thrombocytopenia^*^ |  |  |  |
| Any grade | 27 (31.0) | 25 (48.1) | 0.045 |
| Grade ≥3 | 2 (1.5) | 5 (8.2) | 0.030 |
| Other adverse events |  |  |  |
| Febrile neutropenia | 2 (2.3) | 1 (1.9) | >0.999 |
| Septic shock | 0 | 0 | N/A |
| ICU admission | 0 | 0 | N/A |
| Death | 0 | 0 | N/A |
| Abbreviations: ICU, intensive care unit; N/A, not applicable. ^*^Common Terminology Criteria for Adverse Events (CTCAE) version 5.0. | | | |
